# Determination of Arylcyclohexylamines in Biological Specimens: Sensors and Sample Pre-Treatment Approaches

**DOI:** 10.3390/mi15080984

**Published:** 2024-07-30

**Authors:** Rodrigo Pelixo, Mário Barroso, Eugenia Gallardo, Tiago Rosado

**Affiliations:** 1 Centro de Investigação em Ciências da Saúde, Universidade da Beira Interior (CICS-UBI), 6200-506 Covilhã, Portugal; pelixo.silva@ubi.pt; 2 Laboratório de Fármaco-Toxicologia, UBIMedical, Universidade da Beira Interior, EM506, 6200-000 Covilhã, Portugal; 3 AlphaBiolabs, 14 Webster Court, Carina Park, Warrington WA5 8WD, UK; mbarroso@alphabiolabs.com; 4 Serviço de Química e Toxicologia Forenses, Instituto Nacional de Medicina Legal e Ciências Forenses-Delegação do Sul, 1169-201 Lisboa, Portugal; 5 Centro Académico Clínico das Beiras (CACB)-Grupo de Problemas Relacionados com Toxicofilias, 6200-000 Covilhã, Portugal

**Keywords:** arylcyclohexylamines, toxicology, sample pretreatment, sensors

## Abstract

Arylcyclohexylamine (ACH) compounds represent a predominant faction within new psychoactive substances. Due to their powerful dissociative effects, they are used in recreational contexts but also in situations of drug-facilitated sexual assault, and therefore, they are a constant target of analysis by forensic experts. In recent years, their consumption has been notably high, especially the use of ketamine, presenting daily challenges for laboratories in the determination of this and other ACH analogues. This review comprises the recent strategies that forensic specialists use to identify and quantify ACH compounds in the laboratory with more traditional analytical techniques and technology, and on the point-of-care testing via sensor technology. The study focuses on analogues of phencyclidine (PCP), ketamine, and eticyclidine, highlighting the consistent need for higher sensitivity in the analysis of various samples collected from real cases and simulations of possible matrices. The review also emphasises the ongoing research to develop more sensitive, quicker, and more capable sensors.

## 1. Introduction

New psychoactive substances (NPSs) are considered as any new narcotic that is not controlled by the United Nations but presents harm similar to those listed, according to the European Monitoring Centre for Drugs and Drug Addiction (EMCDDA). Among these, arylcyclohexylamines (ACHs) exhibit a long-standing presence, dating back to the first synthesis of phencyclidine (PCP) in 1956 by Parke-Davis [1]. The powerful central nervous system (CNS) depressant initially started as a potential new anaesthetic; however, it showed strong psychological side effects, including psychotomimetic effects and convulsions, which led to its removal from clinical trials [2]. Some of these same effects formed the basis for the first recreational users of ACHs, leading to the anaesthetic being established as a Schedule II controlled substance by the Drug Enforcement Administration (DEA) [3]. Following these events, the quest for synthesising new ACH compounds gained momentum with the creation of ketamine (KET) in 1962, which presented a more suitable clinical profile compared to its predecessor [1]. Nevertheless, KET also produces psychological effects sought after by recreational users. In the same decade, analogues of PCP in the form of eticyclidine (PCE) were first found on the streets of Los Angeles [4]. ACH compounds represent a public threat due to the potential of these three substances to serve as a basis for new designer drugs—substances that mimic the pharmacology of the originals but with enough structural differences to evade control [1].

**Figure 1:**
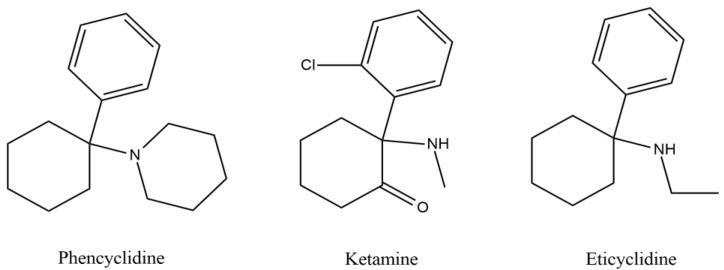
Chemical structure of phencyclidine, ketamine, and eticyclidine.

PCP, the predecessor of the others, is an achiral molecule consisting of three rings: a cyclohexyl group, an aryl group, and an amine group [5]. By adding to or substituting parts of these rings, other ACH compounds are obtained, and slight modifications can easily create new substances (Figure 1). With the prevalence of the cyclohexyl group, these drugs can affect the CNS by binding to the N-methyl-D-aspartate receptor (NMDAr) at the allosteric site, exerting a non-competitive antagonistic activity in the voltage-gated channel. This inhibits Ca^2+^ flux, resulting in a state of dissociative anaesthesia and, in cases of abuse, schizophrenic-like symptoms [5,6].

The number of NPSs based on these three drugs has increased substantially [7]. In 2023, 15 new phencyclidine-type substances were reported worldwide, according to the United Nations Office on Drugs and Crime (UNODC) Early Warning Advisory on NPS-Summary Dashboard. Between 2005 and 2021, 127 new ACH substances were reported in the European Union (EU), and KET alone represented 10% of NPS seizures in the EU in 2021, with 2509 seizures of the dissociative anaesthetic in powder form, where 0.87 tonnes of seized material were KET. This highlights the ongoing prevalence of these substances [8]. For example, in Portugal, significant levels of KET residues were found in the wastewater of two cities: Lisboa, with 19.12 mg per 1000 people per day, and Almada, with 1.91 mg per 1000 people per day [9]. Higher levels were detected in Denmark (Copenhagen, 16.82 mg), Spain (Barcelona, 29.45 mg), and Italy (Milan, 13.82 mg). In Europe, 13% of respondents to the European Web Survey on Drugs reported having consumed KET, with the highest prevalence observed among young adults in Denmark and Romania [10].

The alarming rate at which NPSs, including new ACHs, are synthesised and enter the illicit market required a new strategy in terms of legislation. Although PCE, PCP, and KET are already considered Schedule I, II, and III drugs, respectively [11], the Global Synthetics Monitoring: Analyses, Reporting and Trends (SMART) programme was established in 2008 by the UNODC to report NPSs via online data sharing. In 2014, the EMCDDA published a report on legislation and prosecution, focusing on the legal challenges countries face in combating the rising trend of NPS synthesis. This report provided legal and judicial strategies for policymakers and legal practitioners to apply [12]. Nonetheless, due to the impact of ACHs on society and the current trend of designer drugs, forensic specialists regularly face the challenge of new analogues. Drug designers find new and ingenious ways to slightly alter the chemical structure of ACHs to create new drugs that mimic the effects sought by recreational users, contributing to enormous chemical diversity. These new substances often differ due to changes in metabolic action or observed symptoms, making it difficult to interpret results and assess risks, leaving analysts without clues. In fact, the emergence of new side effects also presents a challenge in emergency services, as it becomes more complicated to determine which treatment or decontamination measures to adopt. Additionally, these changes in the synthesis of new ACHs often result in these substances being present in very low concentrations in biological samples, complicating detection and quantification. The rapid development of new ACHs means that reference standards for many of these substances are not readily available. This drives toxicologists and analysts to develop continuously new methods and update databases to search effectively for these new drugs, as classical methods (e.g., immunoassays) become ineffective and structural libraries of compounds that allow for the verification of the presence of these substances do not exist. In a field where analysis depends on the specific case nature, research on these substances in several biological samples is crucial for successful investigations by the authorities.

This review addresses this last issue related to the determination of these substances and is divided into two parts. The first part focuses on various techniques to extract and determine these analogues in different biological samples, both in research contexts and real case reports. The second part focuses on ongoing efforts to advance on-the-spot investigations of the drugs in question using sensors.

In the first part of the review, a search on the ISI Web of Science database was performed encompassing the combination of keywords “phencyclidine analogues”, “ketamine analogues”, and “eticyclidine analogues” with the biological samples “blood”, “urine”, “oral fluid”, “hair”, “vitreous humour”, “faeces”, “meconium”, “amniotic fluid”, and “teeth”, restricting the search to the last 10 years (from 2013 forward), with the exception of PCE, for which no new results were obtained. The restriction in the search date is due to the large number of existing publications. In some cases, the methodology used in the referenced work was adapted from previous studies, which are also referenced accordingly.

For the second part of the review the ISI Web of Knowledge database was also adopted with the same time restriction, using the keywords “ketamine”, “phencyclidine”, and “eticyclidine” combined with “sensor”. However, due to the lack of studies, the only analyte found was KET in a multitude of matrices. It was decided not to restrict the search to biological samples due to the versatility of situations in which sensors can be used.

## 2. Strategies to Determine Ketamine, Phencyclidine, and Eticyclidine Analogues in Biological Specimens

This first part of the review explores the diverse approaches to identify and quantify ACHs according to biological matrices, specifically blood and its derivatives, urine, and others. The latter includes alternative matrices like hair and oral fluid, as well as less common matrices such as vitreous humour, intraosseous fluid, and samples from nasal swabs. The search reveals a correlation between the number of studies conducted on each matrix and the pharmacokinetics of ACHs in general. These comparisons are examined in more detail through the analysis of tables pertaining to each matrix.

Second, the review focuses on sensor technology to identify KET in a variety of matrices. These new types of solutions are becoming increasingly important to address the ongoing trends of drug abuse, combining material science, organic and inorganic chemistry, and other innovative approaches to develop sensing mechanisms with high sensitivity.

### 2.1. Blood and Derivates

The main routes of administration of ACH are intravenous injection and inhalation, due to poor bioavailability when taken orally in the case of KET, and slow absorption in the case of PCP. The latter is well absorbed by adipose tissue and undergoes enterohepatic circulation, which can extend its presence in the body for up to four weeks [5,13,14]. The necessity of ACHs to circulate in the bloodstream to cross the blood–brain barrier and reach the brain, where they exert activity on NMDAr, justifies the high number and diversity of studies conducted on blood and its derivatives. Table 1 summarizes the various methodologies used to identify and/or quantify multiple analogues in blood, plasma, and serum.

In the first study presented, a validated method was developed to screen and quantify 74 NPSs, including PCP, KET, and their analogues such as 3-methoxyeticyclidine (3-MeO-PCE), 3-methoxyphencyclidine (3-MeO-PCP), 4-methoxyphencyclidine (4-MeO-PCP), and methoxetamine (MXE) [15]. This method integrates fully automated solid-phase extraction (SPE) and liquid chromatography coupled with tandem mass spectrometry (LC-MS/MS), achieving a limit of detection (LOD) of 4 ng/mL for PCP, 0.8 µg/L for KET, and 0.4 ng/mL for the remaining analogues [15]. The limit of quantification (LOQ) for all substances is 5 ng/mL [15]. In a case report, authors linked 3-MeO-PCP to seven deaths in Sweden, identified using liquid chromatography coupled to time-of-flight coupled to mass spectrometry (LC-TOF-MS) after performing a liquid–liquid extraction (LLE) with 0.075% formic acid in acetonitrile and ethanol (90:10 ratio). Quantification was conducted via LC-MS/MS in both whole blood and post-mortem femoral blood. All incidents involved young adults aged 19 to 32 years, each with distinct life circumstances, highlighting the widespread use of these dissociative drugs in the population [16].

In addition to chromatography, researchers and analysts also employ immunoassays to identify ACH compounds. For example, enzyme-linked immunosorbent assays (ELISA) have been utilized for the detection of PCP and its analogues in blood [15,16]. Specific kits for PCP analysis and multi-tests have enabled the detection of substances like 3-MeO-PCP and 3-hydroxyphencyclidine (3-OH-PCP) with high cross-reactivity.

Simplified sample pre-treatment methods have also been integrated into modern analyses. Dried blood spots (DBS) have been used, requiring a low blood volume (100 µL), including *postmortem* blood, where KET and its primary metabolite norketamine (NKET) were screened and quantified using ultra-high pressure liquid chromatography (UHPLC) coupled to MS/MS. Researchers achieved a LOD and LOQ of 0.5 ng/mL and 2 ng/mL, respectively, with a method that involves allowing the sample to dry on a spotting card matrix for two hours, followed by extraction with 0.1% formic acid in methanol [17]. Posterior to validation, the method was applied to 10 *postmortem* samples.

**Table 1:** Sample pretreatment and determination of ACHs and analogues in blood samples and derivatives.

Matrix	Compounds	Sample Amount (μL)	Extraction Method	Screening	Quantification Method	LOD (ng/mL)	LOQ (ng/mL)	Recovery (%)	Reference
Serum	3-MeO-PCE	150	SPE	LC-MS/MS		0.4	5.0	59–79	[15]
	3-MeO-PCP					0.4		61–64	
	4-MeO-PCP					0.4		62–64	
	KET					0.8		74–83	
	MXE					0.4		76–86	
	PCP					4.0		57–74	
Blood	KET	500	DLLME	UHPLC-MS/MS	N.S.	0.5	N.S.	55–86	[18]
	MXE					0.2	N.S.	53–66	
Blood	KET	2000	DLLME	GC-MS		10	10	92–115%	[19]
	NKET					10	50		
	MXE					10	10		
Blood	3-MeO-PCP	200	LLE	LC-MS/MS		0.1	0.3	99	[20]
	4-MeO-PCP					0.1	0.3	81	
	KET					0.3	0.5	95	
	MXE					0.3	0.5	93	
	NKET					0.3	0.5	95	
Plasma	NENK	180	m-SPE	LC-QTOF-MS	N.S.	6	N.S.	82–91	[21,22,23]
Blood	MXE	N.S.	DBS	N.S.	LC-MS/MS	N.S.	N.S.	N.S.	[24]
*Postmortem* Blood	3-MeO-PCP	200	LLE	LC-TOF-MS	LC-MS/MS	N.S.	1.0	N.S.	[25]
Whole blood	3-MeO-PCP	0.5 g	LLE	LC-TOF-MS	LC-MS/MS	N.S.	0.01 mg/g	N.S.	[16,26]
*Postmortem* Blood									
*Postmortem* Blood	3-Meo-PCP	1000	SPE	GC-MS		1	10	N.S.	[27]
Blood	3-MeO-PCP	100	PPT & SPE	LC-QTOF-MS	UHPLC-MS/MS	N.S.	N.S.	N.S.	[28]
Blood	KET	100	DBS	UHPLC-MS/MS		0.5	2	20–45	[17]
	NKET					0.5	2	18–41	
*Postmortem* Blood	KET	100	DBS	UHPLC-MS/MS		0.5	2	47–58	[17]
	NKET					0.5	2	16–26	
Synthetic blood	3-Meo-PCP	N.S.	N.S.	ELISA	N.S.	N.S.	N.S.	N.S.	[29]
	3-OH-PCP								
Femoral Blood	3-MeO-PCP and metabolites	1000	LLE	GC-MS & LC-MS/MS & LC-HRMS	LC-MS/MS	0.05	0.01	97	[30,31]
Blood	KET	500	DLLME	N.S.	UHPLC-MS/MS	0.5	2	87–110	[32]
	NKET					0.05	0.5	72–89	
Blood	2-oxo-PCE	100	LLE	LC-MS/MS		0.22	0.62	76–99	[33]
	DXE					0.15	0.50	79–100	
Blood	PCP	30	N.S.	ELISA	GC-MS	N.S.	N.S.	N.S.	[34]
Blood	3-MeO-PCP	500	LLE	GC -MS & HPLC-UV coupled MS/MS	LC-UV-MS/MS	30	160	>87	[35]
	3-MeO-PCE					50	160		
	MXE					30	160		
Blood	MXPr	N.S.	DLLME	GC-MS	LC-MS/MS	0.5	2	N.S.	[36]
	2F-DCK					0.5	2		
	DXE					0.5	2		
	MXPr			LC-HRMS	N.S.	2	N.S.	N.S.	
	2FDCK					10			
	DXE					10			
Blood	3-Meo-PCP	250 and 500	PPT & SPE	LC-HRAM-Orbitrap-MS & sd-GC-FTIR	N.S.	N.S.	N.S.	N.S.	[37]
Blood	KET	1000	SPE & LLE	GC-MS		N.S.	25	N.S.	[38]
	NET						N.S.		
	DHNK						N.S.		
	PCP						N.S.		
	2FDCK						N.S.		
Serum	3-Meo-PCP	1000	SPE	GC-MS		1	2	>90	[39]
	4-Meo-PCP					N.S.	N.S.	N.S.	
	MXE					N.S.	N.S.	N.S.	
Blood	KET	50	N.S.	CEDIA & DRI & EIA & LC-MS/MS	N.S.	N.S.	N.S.	N.S.	[40,41]
	MXE				LC-MS/MS	0.5	0.5	N.S.	
Blood	KET	1000	SPE	LC-MS/MS		1	5	86–92	[42]
	NKET					1	5	79–85	
Plasma	KET	500	MEPS	GC-MS/MS		5	10	73–89	[43]
	NKET					5	10	63–75	

2FDCK: 2-fluorodeschloroketamine; 2-oxo-PCE: n-ethyl-deschloroketamine; 3-MeO-PCE: 3-methoxyeticyclidine; 3-MeO-PCP: 3-methoxyphencyclidine; 3-OH-PCP: 3-hydroxyphencyclidine 4-MeO-PCP: 4-methoxyphencyclidine; CEDIA: cloned enzyme donor immunoassay; DBS: dried blood spots; DHNK: dehydronorketamine; DLLME: dispersive liquid/liquid microextraction; DXE: deschloroketamine; EIA: enzyme immunoassay; ELISA: enzyme-linked immunosorbent assays; GC: gas chromatography; HPLC: high pressure liquid chromatography; HRAM: high resolution accurate mass; HRMS: high-resolution mass spectrometry; KET: ketamine LC: liquid chromatography; LLE: liquid/liquid extraction; LOD: limit of detection; MEPS: microextraction by packed sorbent; MS/MS: tandem mass spectrometry; m-SPE: magnetic solid phase extraction; MXE: methoxetamine; MXPr: methoxpropamine; NENK: n-ethyl-norketamine; NKET: norketamine; N.S.: non-specified; PCE: eticyclidine PCP: phencyclidine PPT: protein precipitation; QTOF: quantitative time-of-flight; sd-GC-FTIR: solid deposition gas chromatography Fourier transform infrared spectrometry; SPE: solid-phase extraction; TOF: time-of-flight; UHPLC: ultra-high pressure liquid chromatography; UV: ultra-violet.

### 2.2. Urine

The excretion of ACH primarily occurs through urine, while there is a lack of studies on intestinal excretion. This sample is interesting for searching the parent drugs and both active and inactive metabolites; for example, KET is excreted as the parent drug, as well as in the forms of norketamine (NKET), dehydronorketamine (DHNK), and their conjugated metabolites. [14]. Table 2 summarises the methods used to determine ACH in urine. The Department of Forensic Toxicology in New York conducted a study to assess the prevalence of drivers under the influence of ketamine and its analogues in the city. Analysts employed SPE and gas chromatography coupled with mass spectrometry (GC-MS) to screen these compounds in both blood and urine samples. This study marked the first confirmation of positive cases for 2-fluorodeschloroketamine (2FDCK) in the USA, in addition to PCP, KET, and its metabolites.

Following the screening phase, KET quantitation was performed using GC-MS after LLE with n-butyl chloride, followed by acidification and subsequent extraction with toluene, heptane, and isoamyl alcohol [38]. Different sample preparation techniques are frequently applied to enhance existing protocols. For instance, in one study, researchers utilized dual-mode extraction (DME) to reduce both sample volume and the LOD. DME columns facilitate more specific extraction of urine matrix components and include hydrolytic enzymes [44]. Afterwards, the eluent was injected into LC-MS/MS for screening, capable of identifying KET, deschloroketamine (DXE), 2FDCK, and *N*-ethyl-deschloroketamine (2-oxo-PCE) [45]. For these analytes, this method achieved an LOD of 1 ng/mL using only 0.2 mL of sample. In another case, the same LOD was achieved in *postmortem* urine by utilizing 1 mL of sample, which was extracted via SPE and subsequently screened and quantified by LC-MS/MS [42]. This method achieved an LOD of 1 ng/mL and a LOQ of 5 ng/mL for both KET and NKET, with recoveries in the range of 80%. Following validation, this methodology was applied to real samples from cases of unknown deaths in Taiwan, revealing that 11 out of 34 samples tested positive for KET in concentrations ranging from 5 to 23 ng/mL.

In addition to chromatography, other strategies are employed to identify unknown substances. Cross-reactivity had been demonstrated between PCP immunoassays and 3-MeO-PCP, 4-MeO-PCP, and MXE, although MXE showed limitations in detectability. These findings were confirmed using GC-MS, which was previously validated for 3-MeO-PCP. The GC-MS method achieved an LOD of 1 ng/mL, an LOQ of 2 ng/mL, and a recovery rate exceeding 90% [39].

In another specific case, nuclear magnetic resonance (NMR) and single-crystal X-ray diffraction were employed alongside GC-MS and liquid chromatography coupled to high-resolution mass spectrometry (LC-HRMS) to identify a crystalline material seized. This comprehensive study confirmed the presence of a new analogue of KET, namely 2-fluorodeschloro-n-ethyl-ketamine (2FDCNEK). Subsequently, to identify the primary metabolite of this new ACH, a fungal model (*Cunninghamella elegans*) was utilized in metabolism studies, which suggested 2-fluorodeschloro-norketamine as the primary candidate. This finding was later confirmed by HRMS analysis of urine from an individual who had abused the new substance, 2FDCNEK [46].

**Table 2:** Sample pretreatment and determination of ACHs and analogues in urine samples.

Compounds	Sample Amount (µL)	Extraction Method	Screening	Quantification Method	LOD (ng/mL)	LOQ (ng/mL)	Recovery (%)	Reference
2-oxo-PCE	4000	LLE	GC-MS & LC-MS/MS	N.S.	N.S.	N.S.	N.S.	[47,48]
3-Meo-PCP	250 and 500	PPT & SPE	LC-HRAM-Orbitrap-MS & sd-GC-FTIR	N.S.	N.S.	N.S.	N.S.	[37]
KET	1000	SPE/LLE	GC-MS		N.S.	25	N.S.	[38]
NKET					N.S.	N.S.	N.S.	
DHNK					N.S.	N.S.	N.S.	
PCP					N.S.	N.S.	N.S.	
2FDCK					N.S.	N.S.	N.S.	
KET	N.S.	N.S.	LC-MS/MS		N.S.	N.S.	N.S.	[49]
DXE								
KET	1600	SPE	Immunoassay & GC-MS/MS & LC-MS/MS	N.S.	4	N.S.	N.S.	[50,51]
DXE					N.S.	N.S.	N.S.	
2-oxo-PCE					N.S.	N.S.	N.S.	
3-Meo-PCP					N.S.	N.S.	N.S.	
3-OH-PCP					N.S.	N.S.	N.S.	
KET	200	DME	LC-MS/MS	N.S.	1	N.S.	48	[45]
DXE					1	N.S.	50	
2FDCK					1	N.S.	51	
2-oxo-PCE					1	N.S.	55	
2FDCK and metabolites	1000	SPE	UHPLC-QTOF-MS & UHPLC-MS/MS	N.S.	N.S.	N.S.	N.S.	[48,52]
KET					5	N.S.	N.S.	
DXE					N.S.	N.S.	N.S.	
2-oxo-PCE					N.S.	N.S.	N.S.	
Tiletamine					5	N.S.	N.S.	
3-Meo-PCP	1000	SPE	Immunoassay’s & GC-MS	GC-MS	1	2	>90	[39]
4-Meo-PCP					N.S.	N.S.	N.S.	
MXE					N.S.	N.S.	N.S.	
KET	50	N.S.	CEDIA & DRI & EIA & LC-MS/MS	LC-MS/MS	N.S.	N.S.	N.S.	[40,41]
MXE					0.5	N.S.	N.S.	
KET	1000	SPE	LC-MS/MS		1	5	86–92	[42]
NKET					1	5	79–85	
2FDCNEK	2000	LLE	CG-MS & NMR & single-crystal X-ray diffraction & LC-HRMS	N.S.	N.S.	N.S.	N.S.	[46]
KET	2000	DLLME	GC-MS		5	10	92–115	[19]
NKET					10	50		
MXE					10	10		
MXE	N.S.	N.S.	N.S.	LC-MS/MS	N.S.	N.S.	N.S.	[24]
3-MeO-PCP and metabolites	1000	LLE	GC-MS & LC-MS/MS & LC-HRMS	LC-MS/MS	0.05	0.01	97	[30,31]
3-MeO-PCP	500	LLE	GC-MS & HPLC-UV-MS/MS	HPLC-UV-MS/MS	20	160	>98%	[35]
3-MeO-PCE					10	160		
MXE					40	160		
KET	500	MEPS	GC-MS/MS		5	10	89–101	[43]
NKET					5	10	73–77	

2FDCK: 2-fluorodeschloroketamine; 2FDCNEK: 2-fluorodeschloro-n-ethyl-ketamine; 2-oxo-PCE: n-ethyl-deschloroketamine; 3-MeO-PCE: 3-methoxyeticyclidine; 3-MeO-PCP: 3-methoxyphencyclidine; 3-OH-PCP: 3-hydroxyphencyclidine 4-MeO-PCP: 4-methoxyphencyclidine; CEDIA: cloned enzyme donor immunoassay; DHNK: dehydronorketamine; DLLME: dispersive liquid/liquid microextraction; DME: dual mode extraction; DXE: deschloroketamine; EIA: enzyme immunoassay; GC: gas chromatography; HPLC: high pressure liquid chromatography; HRAM: high resolution accurate mass; HRMS: high resolution mass spectrometry; KET: ketamine; LC: liquid chromatography; LLE: liquid/liquid extraction; LOD: limit of detection; MEPS: microextraction by packed sorbent; MS/MS: tandem mass spectrometry; MXE: methoxetamine; NKET: norketamine; NMR: nuclear magnetic resonance; N.S.: non-specified; PCP: phencyclidine; PPT: protein precipitation; QTOF: quantitative time-of-flight; SPE: solid-phase extraction; sd-GC-FTIR: solid deposition gas chromatography Fourier transform infrared spectrometry; UHPLC: ultra-high pressure liquid chromatography; UV: ultra-violet.

### 2.3. Other Specimens

Table 3 describes the analysis of ACHs in alternative matrices, specifically hair, vitreous humour, intraosseous fluid, oral fluid, and nasal swab. Alternative matrices offer several advantages compared to traditional matrices like blood and urine: they are non-invasive, easy to collect, and in some cases, highly stable, allowing for analysis even in autopsies where the body may have significantly deteriorated.

In the case of autopsies, researchers developed a method using ultra-high-pressure liquid chromatography coupled with high-resolution mass spectrometry (UHPLC-HRMS) following methanolic incubation, which enabled detection of MXE, KET, and NKET in 30 mg of hair with an LOD of 5 pg/mg and 10 pg/mg for the latter two, respectively. The value for MXE was not reported. This method was subsequently applied to 300 forensic cases [53]. This method enabled the construction of a database containing over 170 analytes, including various drugs of abuse and pharmaceutical substances.

With regard to the use of hair as a matrix, it is important for researchers to follow the established guidelines by the Society of Hair Testing. Several steps, from decontamination procedures, crucial steps to the sample preparation protocol, and cut off values that prove the presence of the drug are described. If these criteria are not meet, the method is not eligible to confirm positive cases. In this context, the only ACH with a cutoff defined is KET, with the value of 200 pg/mg and the presence of NKET definitely proves the consumption of KET. Other ACH information is not described [54].

With the aim of analysing *postmortem* samples, analysts developed and validated a methodology to determine 3-MeO-PCP, 3-MeO-PCE, and MXE in blood, urine, and vitreous humour. The vitreous humour sample was simulated with water, and matrix interferences were assessed by spiking three blank samples of porcine vitreous humour.

Using LLE and GC-MS, as well as LC-MS/MS, researchers achieved an LOD of 0.05 mg/L for 3-MeO-PCP and 0.02 mg/L for 3-MeO-PCE and MXE. The LOQ for all analogues was 0.16 mg/L, with a recovery rate exceeding 98%, using 0.5 mL of sample [35]. This is an interesting approach; in this work an HPLC–UV system interfaced with an MS/MS equipped with an electrospray ionisation source (ESI) was used. The authors demonstrated that HPLC–UV is a robust, accurate, and precise method for the qualitative and quantitative analysis of these substances in biological specimens, while the mass spectrometer serves as a useful confirmatory tool. Another uncommon matrix of special interest for *postmortem* analysis is intraosseous fluid. Similar to vitreous humour, this fluid is less compromised than blood in *postmortem* situations, a characteristic upon which researchers based their study. They compared peripheral and cardiac blood with fluid collected from the tibia and humerus by drilling into the region with a needle to access the zone of interest and applying suction with a syringe for 5–10 min. Following collection, drug screening with ELISA was performed on both samples, alongside GC-MS analysis on cardiac blood to confirm and quantify PCP. This was necessary due to the insufficient volume of intraosseous fluid collected [34]. Oral fluid is often used as an alternative matrix due to its ease of collection and non-invasiveness. In the case of ACHs, oral consumption is not common due to the low bioavailability of these substances [14]. However, the visual similarity to other drugs is high, which can lead to mistaken consumption by substance abusers.

Focusing on this alternative matrix, two groups of researchers developed distinct methods to identify ACHs. Bianchi et al. [55] utilized microextraction by packed sorbent (MEPS) to extract KET from 400 μL of sample. KET was then screened and quantified using desorption electrospray ionization (DESI) coupled with HRMS and confirmed by GC-MS [55]. For DESI, polytetrafluoroethylene, silica-based, and polylactide-based materials were tested as substrates, with the latter showing the best performance for the determination of the analytes in question, achieving an LOQ of 50 ng/mL.

Oral fluid is one of the matrices with guidelines established by the Health and Human Services and the Substance Abuse and Mental Health Services Administration. The “Mandatory Guidelines for Federal Workplace Drug Testing Programs” describe the drugs, analytes, cutoffs, collection procedures, and other considerations for sample analysis. In the document, PCP is the only ACH considered, with a cutoff of 10 ng/mL. Federal organizations, as well as others, must follow these guidelines to confirm drug presence [56].

The other group developed a molecularly imprinted polymer (MIP) using 3-OH-PCP as a template, which was used as a sorbent in SPE to extract the hydroxy analogue of PCP. Ion mobility spectrometry (IMS) was employed to determine the analyte, and this method achieved an LOD of 15 ng/mL, an LOQ of 50 ng/mL, and a recovery of 70% with 1 mL of sample [57].

**Table 3:** Sample pretreatment and determination of ACH and analogues in other biological samples.

Matrix	Compounds	Sample Amount	Extraction Method	Screening	Method Quantification	LOD	LOQ	Recovery (%)	Reference
Hair	KET	7 g	PLE & DLLME	HPLC-HRMS/MS		2 pg/mg	5 pg/mg	60	[58]
	MXE					1 pg/mg	25 pg/mg	52	
	PCP					1 pg/mg	25 pg/mg	48	
Water to replicate the vitreous humour	3-MeO-PCP	500 μL	LLE	GC -MS & HPLC-UV coupled ESI-MS/MS	HPLC-UV coupled MS/MS	50 ng/mL	160 ng/mL	>98	[35]
	3-MeO-PCE					20 ng/mL	160 ng/mL		
	MXE					20 ng/mL	160 ng/mL		
Intraosseous fluid from tibia and humorous	PCP	30 μL	N.S.	ELISA	GC-MS	N.S.	N.S.	N.S.	[34]
Hair	KET	50 mg	LLE	UHPLC-MS/MS		N.S.	N.S.	N.S.	[59,60]
Hair	KET	30 mg	LLE	UHPLC-HRMS	N.S.	5 pg/mg	N.S.	N.S.	[53]
	MXE					N.S.	N.S.	N.S.	
	NKET					10 pg/mg	N.S.	N.S.	
Hair	KET	21 and 50 mg	LLE	LC-HRMS	N.S.	5 pg/mg	N.S.	N.S.	[61]
	MXE					5 pg/mg	N.S.	N.S.	
Hair	MXPr	30 mg	LLE	GC-MS	LC-MS/MS	0.01 ng/mg	0.05 ng/mg	N.S.	[36]
	2FDCK					0.01 ng/mg	0.05 ng/mg	N.S.	
	DXE					0.01 ng/mg	0.05 ng/mg	N.S.	
	MXPr			LC-HRMS	N.S.	0.05 ng/mg	N.S.	N.S.	
	2FDCK					0.05 ng/mg	N.S.	N.S.	
	DXE					0.05 ng/mg	N.S.	N.S.	
Hair	2FDCK	20 mg	LLE	Immunoassays & LC-HRMS & LC-MS/MS & HS-GC- FID	LC-HRMS	N.S.	N.S.	N.S.	[62,63]
	3-MeO-PCE					N.S.	N.S.	N.S.	
Hair	KET	50 mg	MEPS	GC-MS/MS		0.01 ng/mg	0.05 ng/mg	39–60	[64]
	NKET					0.05 ng/mg	0.05 ng/mg	32–43	
Nasal swab	2-oxo-PCE	N.S.	N.S.	HPLC-DAD & IT-TOF/MS	N.S.	N.S.	N.S.	N.S.	[47,65]
Oral fluid	KET	400 μL	MEPS	GC-MS & DESI-HRMS		N.S.	50 ng/mL	99–120	[55]
Oral fluid	3-OH-PCP	1000 μL	MIP-SPE	IMS		15 ng/mL	50 ng/mL	70	[57]

2FDCK: 2-fluorodeschloroketamine; 2-oxo-PCE: n-ethyl-deschloroketamine; 3-MeO-PCE: 3-methoxyeticyclidine; 3-MeO-PCP: 3-methoxyphencyclidine; 3-OH-PCP: 3-hydroxyphencyclidine; DAD: diode array detector; DESI: desorption electrospray ionization; DLLME: dispersive liquid/liquid microextraction; DXE: deschloroketamine; EI/CI: ion trap electron and chemical ionization; ELISA: enzyme-linked immunosorbent assays; ESI: electrospray ionization; FID: flame ionization detector; GC: gas chromatography; HPLC: high pressure liquid chromatography; HRMS: high resolution mass spectrometry; HS: headspace; IMS: ion mobility spectrometry; IT: ion trap; KET: ketamine; LC: liquid chromatography; LLE: liquid/liquid extraction; LOD: limit of detection; MEPS: microextraction by packed syringe; MIP: molecularly imprinted polymer; MS/MS: tandem mass spectrometry; MXE: methoxetamine; MXPr: methoxpropamine; NKET: norketamine; N.A.: non-applicable; N.S.: non-specified; PCP: phencyclidine; PLE: pressurized liquid extraction; TOF: time-of-flight; UHPLC: ultra-high pressure liquid chromatography; UV: ultra-violet.

The forensic approach to biological samples is determined based on the nature of the case. For instance, blood and urine samples are collected when analysts need to establish intoxication at the time of occurrence, such as in cases of acute intoxication or fatalities. Blood is the primary choice for quantitative analysis because it directly correlates with the clinical status of the individual. In the case of blood samples and their derivatives, considering the information presented in Table 1, the volume typically used is less than 0.5 mL. In both *antemortem* and *postmortem* situations, peripheral blood is commonly used, with particular importance in *postmortem* cases to avoid contamination due to redistribution phenomena. Urine, on the other hand, often contains a higher concentration of the substance and has fewer interferences than blood; however, it is only present in high concentrations in the later stages after consumption [66]. In this case, due to the greater availability of the sample, it is common to use larger volumes (2 mL or more) during the sample pre-treatment process. One of the main limitations of this sample is the presence of metabolites, many of which are unknown due to the lack of knowledge about the metabolic patterns of new ACH. This complicates the interpretation of intoxication scenarios.

Alternative samples serve a less direct purpose. Hair analysis is used to detect less recent or recurring consumption, particularly in fragmentation analysis, due to its wide detection range. The only way to remove this record is by cutting the hair, but there is no direct correlation between the dose consumed and the amount detected in the hair, which is a complex matrix with many potential interferences [67]. Hair samples are also useful in cases of drug-facilitated sexual assault, where certain derivatives of NPS may be used for this purpose, such as KET. There is more information available specifically on this substance. The Society of Hair Testing recommends a cutoff value of 0.2 ng/mg to identify KET consumption, as well as the presence of the metabolite NKET (although no established cutoff is set for NKET). Usually, the sample amount is between 10 to 50 mg of hair. Oral fluid allows for the detection of substances shortly after consumption and is therefore difficult to adulterate by the user [68]. However, because oral consumption of ACH is uncommon, a nasal swab is often more useful for detecting remnants of substances in the nasal cavity.

Other samples, such as intraosseous fluid or vitreous humour, exploit the *postmortem* redistribution effect to detect the presence of substances during the later stages of body decomposition. Vitreous humour is particularly useful for detecting substances in advanced stages of putrefaction, although there is no correlation between detection and consumption dose [69].

**Figure 2:**
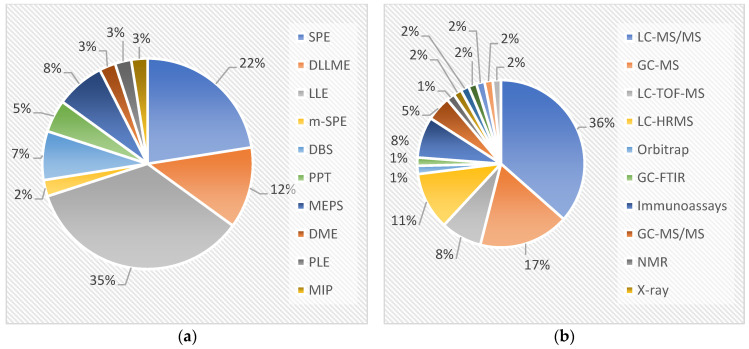
Methods used in ACH analysis: (**a**) extraction methods, and (**b**) analytical instrumentation.

In forensics, the primary concern regarding substance determination is the method’s ability to detect, and when necessary, quantify the analytes present. To achieve maximum sensitivity and selectivity, forensic analysts select and refine methods to minimise sample interferences and enhance analyte specificity. Figure 2 statistically represents the prevalence of the extraction and determination methods described in this manuscript.

Sample pre-treatment is a crucial step in the process; however, performance is not the only criterion. In this study, 35% of the methods employed LLE as the extraction technique (Figure 2a), which often allowed for low LODs and LOQs. In addition to its good performance, LLE is an easy and relatively fast technique, preferred for routine laboratory use, which explains its high utilisation. However, the need for large quantities of organic solvents raises environmental concerns. One of the main limitations of this technique is the lack of automation, the formation of emulsions, and the extraction of lipids alongside the analytes of interest, particularly in blood and derivative samples, which can pose problems when injected into GC-MS. SPE is a technique where extraction efficiency depends on the interaction between the sample and the stationary phase and solvents used in the elution process. It is also widely used due to its automation potential; indeed, some analytical equipment manufacturers have developed online solutions to facilitate this aspect. However, SPE is still outperformed by LLE in terms of recovery, and cartridges must be discarded after a few uses due to low reproducibility. To achieve even lower solvent consumption, greener extraction techniques have recently gained popularity. For example, dispersive liquid–liquid microextraction (DLLME) focuses on the dispersion of extraction solvent within the aqueous sample and is considered a refined approach to LLE. Another technique, microextraction by packed sorbent (MEPS), was developed to miniaturise SPE and allow for greater cartridge reuse. However, both techniques have drawbacks: MEPS is characterised by low recoveries, and DLLME involves complex steps, which likely explains their lesser use [70,71].

The analytical approach to substance determination defines the ability to identify ultimately and quantify the substance of interest. As presented in the figure, chromatography is by far the most common solution, with a particular emphasis on LC-MS/MS, which accounts for 36% of the analyses (Figure 2b) due to the high separation capacity of liquid chromatography and the high sensitivity of mass-to-charge ratio detection. GC-MS is the second most used method, relying on the separation based on the volatility of compounds and, likely, its more affordable cost compared to LC-MS/MS systems. However, one drawback is that some ACH analogues require derivatisation to increase sensitivity and, consequently, detection capability in samples from intoxication scenarios. Immunoassays are often used in forensic laboratories as screening methods due to their ease of use, although they require more complex techniques for detection. Nevertheless, for most ACH analogues, commercial kits are not available. It is also necessary to verify cross-reactions to avoid false positives and false negatives.

### 2.4. Sensors

In this second approach, the study focuses on the emerging analytical trend of sensors. These devices offer ease of manoeuvrability, rapid analysis times, high sensitivity, and relatively low cost. In some cases, they can even facilitate point-of-care analysis by experts or be constructed as “do-it-yourself” (DIY) devices for ease of use by potential victims in cases of suspicion. Another advantage of sensor technology is its suitability for use in on-the-spot investigations. Due to these characteristics, sensor development has greatly expanded with advancements in nanotechnology and diversified applicability.

Herein, the study does not limit itself to biological matrices, as this would not do justice to the wide range of circumstances in which these devices can be applied. Accordingly, in addition to the matrices covered in the first segment of the study, other non-biological samples have become targets for various sensor technologies. These include solid samples such as seized materials, as well as alcoholic and non-alcoholic beverages, which are often spiked by sex offenders. Table 4 describes this broad applicability of sensor technology for analysing KET.

Fluorescent approaches are among the most common methods used to detect illicit drugs via sensors, utilizing chemical mechanisms that react with the analyte and emit fluorescence. In the case of the first sensor [72], researchers developed a multi-colour fluorescent carbon dots (CDs) probe in solution. When added to a KET solution, this probe emits fluorescence within 5 min. This method employs a dual-mode strategy, allowing detection with the naked eye and drug discrimination via fluorescence spectrometry, achieving a detection limit of 1 ng/mL.

Furthermore, the researchers applied deep learning models to improve continually detection capabilities, with potential future applications in forensic scenarios involving urine samples [72].

Another dual-mode approach to identify KET, specifically in the hydrochloride form in soda drinks, was developed to detect spiked beverages in cases of assault. This method utilized optical techniques including colorimetry via gold nanoparticles (NP) and CDs. The approach relied on a quenching mechanism where, in the absence of KET, the dispersed particles exhibited a red colour and no fluorescence. However, in the presence of the analyte, the particles aggregated, changing the colour to blue and allowing the CDs to cease quenching. Both methods achieved a detection limit in the range of 10^−5^ mol/L, although they encountered challenges in orange-coloured drinks [73]. This method allows the drink collected from the crime scene to be analysed by adding an aliquot of it to the sensor solution, enabling a rapid forensic analysis.

Luminogens are commonly used in optical sensors, but besides AuNPs and CDs, other strategies exist. In a study [74], researchers utilized the quenching properties of cucurbit[8]uril (Q8) and the fluorescent properties of palmatine to develop an optical sensor capable of detecting 5 ng/mL of KET in artificial urine. Chemically, by adding twice the concentration of palmatine to the Q8 solution, palmatine dimerizes within the barrel-shaped cavity of Q8, resulting in a quenching effect. In the presence of KET, palmatine solubilises because it binds weakly to Q8, thereby restoring luminescence.

Besides luminescence sensors, some optical sensors utilize terahertz (THz) frequency to detect analytes. Monir et al. [75] developed a hexagonal photonic crystal fibre for detection of KET in blood. This structure consists of five layers of air holes and hexagonal core areas. The drug is injected into the centre of the circle, and a laser incident on the fibre enables analysis of X and Y polarizations in the THz range, allowing for discrimination. This method achieved an optimal relative sensitivity of 90%.

Electrochemical sensors are also at the forefront of sensor technology, employing diverse techniques and materials to develop more sophisticated and sensitive electrodes for identifying analytes. Jin et al. [76], combined MIP technology with L-cysteine membranes to create a new sensor using KET as a template for detection of ACH in hair and urine. After polymerization, cyclic voltammetry (CV) and square wave voltammetry (SWV) characterized the sensor, revealing that the KET electrochemical behaviour is influenced by adsorption. The membrane demonstrated sensitivity to analyte accumulation, achieving an LOD of 1.6 × 10^−7^ M within 10 min of analysis. The sensor also showed recovery rates of 95–101% in hair and 93–102% in urine. These features demonstrate the sensor’s capability to detect KET abuse following transgressions, as real samples from drug users were collected to assess the practical application of the developed sensor. Molecular complexes between organic and inorganic compounds represent a strategy with high optimization potential in sensor development, influenced by factors such as pH effects, solvent proportions, choice of plasticizers, and others. El-Naby [77] developed multiple membranes to optimize these parameters, including a-Keggin polyoxomolybdophosphate:18-crown-6 (PM-CE), PM-CE/potassium tetrakis(p-chlorophenyl)borate (KTpClPB), phosphomolybdic–ketamine (PM-KT) ion pair, and 18-crown-6 (CE). They tested the sensitivity of these proposed membranes in PVC plasticizer with commercially available pharmaceutical doses of KET. PM-CE/KTpClPB exhibited the highest sensitivity with an LOD of 7.9 × 10^−7^ M.

As previously mentioned, ACHs are commonly used as spiking substances in cases of sexual assault, making any form of protection for victims invaluable. With this goal in mind, researchers developed a wearable electronic finger using DIY fabrication to help victims protect themselves before any assault of this matter [78]. The sensor utilized 3D printed electrodes made from polylactic acid and was calibrated in spirit drinks spiked with KET. In real-time, the sensor achieved LODs of 0.1 mM in whiskey, vodka, and gin and 0.2 mM in beer. The analysis results are immediately uploaded to a smartphone. This low-cost and easy-to-fabricate sensor offers a potential solution for preventing sexual assault incidents by allowing the user to simply dip the e-finger into the suspected spiked drink. The discreet result upload feature helps to avoid raising the aggressor’s suspicion and putting the victim at further risk.

The advances of nanocomposites have significantly impacted material science, particularly in the realm of sensor technology. One recent trend involves the development of sensors for detecting KET in plasma using functionalized multiwall carbon nanotubes@gold nanoparticles (f-MWCNTs@AuNP) nanocomposites embedded in a sol-gel matrix on a pencil graphite electrode (PGE) [79]. This sophisticated system achieved detection of KET at a concentration of 0.7 nM in 2 mL of plasma within 7 min. To enhance sensor performance, the nanocomposite underwent thorough characterization using X-ray diffraction, transmission electron microscopy, X-ray analysis, and various electrochemical techniques such as CV, SWV, and electrochemical impedance spectroscopy (EIS). The sensor maintained its sensitivity over two weeks of testing, retaining 93% of the original current measurements after this period.

In addition to potentiometry, electrochemical sensors utilize a range of parameters. In the study of Sadrolhosseini et al. [80], researchers developed a plasmonic sensor using a nanocomposite of polyaniline-reduced graphene oxide (PANI-rGO) and iron oxide, integrated into a surface plasmon resonance (SPR) setup. This sensor demonstrated the capability to detect KET at concentrations as low as 0.1 ppm within 220 s. Such analyses confirm the sensitivity of nanocomposites in sensor applications, showcasing their potential as versatile sensing layers for detecting KET and potentially other substances.

Less common than optical and electrochemical sensors, but still effective, are electronic nose sensors. In this research, only one study was identified with this type of sensor. The authors [81] proposed a system capable of detecting KET in gas and cigarette smoke. This system comprises a pump, a flow control valve, a sensor chamber equipped with five Figaro TGS sensors and associated circuitry, a data acquisition card, and a laptop for data analysis. The Figaro sensors in the presence of oxygen have high resistance. When a reducing gas (in this case KET gas) is present in the atmosphere, it reacts with the oxygen, decreasing resistance leading to an increase in current and signal emission. To evaluate the system’s performance, the researchers collected 1 L of KET cigarette smoke, KET gas, and combinations of both into a bag. They measured and plotted the rate of resistance variation for different concentrations. The method achieved an accuracy of 96%, with a false positive and false negative rate of 2% for both scenarios. This electronic nose system demonstrates promising potential for detecting KET in gas and cigarette smoke, offering a unique approach within sensor technology for substance detection. This enables police officers to collect air samples in 1 L bags when they detect the odour of a KET cigarette or KET gas (odour produced by the heating of KET cigarettes, which is KET mixed with tobacco or marijuana in cigarettes). In the case of positive results, they can then obtain an appropriate warrant for the location in question.

**Table 4:** Sensor technology to identify KET.

Matrix	Compounds	Sample Amount	Type of Sensor	Transducing Mechanism	Sensing Mechanism	LOD	Time of Analysis	Reference
Solution	KET	N.S.	Optical	Fluorescence	Multi-colour fluorescent carbon dots	1 ng/mL	5 min	[72]
Oral fluid	KET	N.S.	Electrochemical	Potentiometry	Unmodified graphite screen printed electrodes	1.1 µM	10 min	[82]
Urine	KET	0.1 mL	Optical	Colorimetry	DNA-AuNP’s	N.S.	N.S.	[83]
Solution	KET	N.S.	Optical	THz frequency	Porous core PCF	N.S.	N.S.	[84]
Aqueous solution	KET	0.005 mL	Optical	Solid-state fluorescence	8Ba-2f fluorescent probe on filter paper	50 pg/cm^2^	Real-time	[85]
Whiskey	KET	20 mL	Electrochemical	Potentiometry	Polylactic acid electrodes	0.14 mM	Real-time	[78]
Vodka						0.11 mM		
Gin						0.13 mM		
Beer						0.18 mM		
Water vapor	KET	N.S.	Optical	Fluorescence	PBI-CB	N.S.	1 min and 10 s	[86]
Saliva	KET	N.S.	Optical	Reflected light	Antibody combined with magnetic nanobeads	100 ng/mL	120 s	[87]
Hair	KET	N.S.	Optical	Fluorescence	Up-converting nanoparticles	1 ng/mL	5 min	[88]
Saliva	KET	N.S.	Electrochemical	Potentiometry	Aptamer conjugated with gold electrodes	10 nM	30 s	[89]
Urine								
Oral fluid	KET	N.A.	Optical	Colorimetry	Competitive paper-based ELISA	0.03 ng/mL	6 min	[90]
Soda drinks	KET	N.S.	Optical	Colorimetry	AuNP’s	2.70 × 10^−5^ M	N.S.	[73]
			Optical	Fluorescence	Fluorescent CDs	1.33 × 10^−5^ M	N.S.	
Serum	KET	2 mL	Optical	Fluorescence	Fluorescein tagged with Y-shape DNA and AuNPs	3 pg/mL	1.5 h	[91]
Hair	KET	10 mg	Electrochemical	Electrochemiluminescence	AuNP’s/Indium tin oxide via antibody recognition	5.73 pg/g	30 min	[92]
Soft beverages	KET	Single aliquot	Electrochemical	Potentiometry	Polyaniline nano-dispersion	3.2 × 10^−6^ M	20 s	[93]
			Optical	Fluorimetry	CD/AuNP’s	2 × 10^−4^ M	1 min	
				Colorimetry	Co(SCN)2	10 mg/mL	Instant	
Urine	KET	N.S.	Electrochemical	Resonance frequency	KET antibody	0.86 pg/mL	10 min	[94]
Pepsi	KET	0.1 mL	Optical	Colorimetry	Bromocresol green	N.S.	5 s	[95]
Rum						N.S.	N.S.	
Whiskey						N.S.	N.S.	
Urine	KET	1 mL	Electrochemical	Chemiluminescence	Lu + NBS	N.S.	N.S.	[96]
					Lu+; K_3_Fe[CN]_6_	N.S.	N.S.	
					Cal + NBS	N.S.	N.S.	
					Cal + KMnO_4_	N.S.	N.S.	
Artificial urine	KET	N.S.	Optical	Fluorescence	Palmatine within the cavity of Q8	5.0 ng/mL	30 s	[74]
Solution	KET	10 mL	Electrochemical	Potentiometry	PM-CE	2.6 × 10^−6^ M	60–10 s	[77]
					PM–CE/KTpClPB	7.9 × 10^−7^ M	50–10 s	
					PM-KT	1.3 × 10^−5^ M	60–20 s	
					CE	4.5 × 10^−5^ M	90–40 s	
Urine	KET	N.S.	Electrochemical	Potentiometry	Carbon paste electrode	7.3 × 10^−6^ M	8–18 s	[97]
Ampoules								
Urine	KET	5 mL	Electrochemical	Potentiometry	PM-KT, Na-TPB, and DBP electrode	1.2 × 10^−7^ M	7 s	[98]
Whiskey	KET	µL range	Electrochemical	Potentiometry	Zeo-GO	0.001 nm/mL	2 s	[99]
Real Juice								
Urine								
Blood	KET	1.5 mL	Optical	Fluorescence	DNA-templated silver nanoclusters probes	0.06 ng/mL	5 min	[100]
Serum	KET	N.S.	Electrochemical	Potentiometry	Au electrode modified by 3-MPA, EDC and NHS with KET antibody	0.41 × 10^−6^ µM	N.S.	[101]
Whiskey	KET	0.01 mL	Electrochemical	Admittance	Au interdigitated electrodes	0.16 ng/μL	N.S.	[102]
				Capacitance		0.73 ng/μL		
Plasma	KET	2 mL	Electrochemical	Potentiometry	PTY, sol-gel, f-MWCNTS@AuNPS on a PGE	0.7 nM	420 s	[79]
Urine	KET	N.S.	Electrochemical	Potentiometry	MAA and EGDMA on a MOFS@G modified screen-printed electrode	4.0 × 10^−11^ M	5 min	[103]
Powdered illicit drugs	KET	20–50 mg	Optical	Colorimetric	Co(SCN)2 into PDMS	100 µg/ml	10 min	[104]
Cigarette gas	KET	1000 mL	Electronic nose	Resistance variation rate	Figaro TGS metal oxide	N.S.	3 min 20 s	[81]
Gas								
Hair	KET	20 mg	Electrochemical	Potentiometry	Poly-L-cysteine molecular imprinted membrane	1.6 × 10^−7^ M	10 min	[76]
Urine		9 mL						
Blood	KET	N.S.	Optical	Thz frequency	Hexagonal photonic crystal fibre	N.S.	N.S.	[75]
Powder	KET	5–10 mg	Electrochemical	Photoluminescence	TA-Ag NCs	0.16 mM	N.S.	[105]
Sewage	KET	10 mL	Electrochemical	Potentiometry	Fe_3_O_4_ @MIPs/magnetic glassy carbon electrode	8.0 × 10^−13^ M	4 min	[106]
Controlled drugs	KET	N.S.	Optical	Visible and infrared frequency	PCF	N.S.	N.S.	[107]
Plasma	KET	1 mL	Electrochemical	Potentiometry	TMSPMA modified Si-NPs MIP on polyaniline modified carbon screen-printed electrode	9.29 × 10^−7^ M	3–5 s	[108]
Urine								
Beverages								
Solution	KET	N.S.	Electrochemical	SPR	PANI- rgo-Fe_3_O_4_ nanocomposite	0.1 ppm	220 s	[80]
Powder	KET	0.05 mL	Electrochemical	Potentiometry	Carbon-based screen-printed electrodes	N.S.	60 s	[109]
Crystals								

3-MPA:3-mercaptopropionic; 8BA-2F: ((((Hexane-1,6-diylbis(2,7-bis(4-(hydroxymethyl)-phenyl)-9H-fluorine-9,9-diyl))bis(hexane-6,1-diyl))bis-(9H-carbazole-9,3,6-triyl))tetrakis(benzene-4,1-diyl))tetramethanol; Cal: calcein; CD: carbon dots; CE: 18-crown-6; DBP: dibutyl phthalate; DNA: deoxyribonucleic acid; EDC: 1-ethyl-3-(3-dimethylaminoprophyl) carbodiimide; EGDMA: ethylene glycol dimethacrylate; EIS: electrochemical impedance spectroscopy; ELISA: enzyme-linked immunosorbent assays; f-MWCNTs@AuNP: functionalized multiwall carbon nanotubes@gold nanoparticles; KET: ketamine; KTpClPB: potassium tetrakis(p-chlorophenyl)borate; Lu: luminol; MAA: methacrylic acid; MIP: molecularly imprinted polymer; MOFs@G: metal–organic framework/graphene nanocomposite; Na-TPB: lipophilic anionic additive; NBS: n-bromosuccinimide; NHS: n-hydroxysulfsuccinimide; NP: nanoparticles; N.S.: non-specified; PANI-rGO: polyaniline-reduced graphene oxide; PBI-CB: o-carborane derivative of perylene bisimide; PCF: photonic crystal fibres; PDMS: polydimethylsiloxane; PGE: pencil graphite electrode; PM-CE: a-keggin polyoxomolybdophosphate:18-crown-6; PM-KT: phosphomolybdic–ketamine; PTY: polytyramine; Q8: cucurbit[8]uril; SPR: surface plasmonic resonance; TA-Ag NCs: thiosalicylic acid-stabilized silver nanoclusters; THz: terahertz; TMSPMA: 3-(trimethoxysilyl)propyl methacrylate; Zeo–GO: zeolites nanoflakes and graphene-oxide nanocrystals.

## 3. Conclusions

In recent years, ACH use has increased in both recreational and medical contexts. The popularity of KET and its analogues, like MXE, has grown, particularly in recreational settings. New PCP analogues, including 3-MeO-PCP and 4-MeO-PCP, also mimic PCP effects and are being increasingly identified in forensic analyses due to their potential misuse.

From a toxicological perspective, ACH analogues pose significant challenges: (1) Identification and detection are complicated by the constant emergence of new analogues and their varied metabolism, requiring extensive research. (2) Toxic effects can be severe and unpredictable. Their rising popularity and easy availability through unregulated markets increase overdose risks and adverse effects, exacerbated by inadequate regulation. Regarding their therapeutic use, KET and its analogues show promise in treating depression, post-traumatic stress disorders, and severe anxiety, though their use is monitored due to potential side effects and abuse risks. Research continues to balance their benefits with safety concerns, highlighting the unique NMDA receptor antagonism of KET compared to traditional medications.

The increased availability and consumption of these substances have led to a greater focus on their effects and the need for comprehensive guidelines and controls. Forensic experts are continuously monitoring and analysing these substances to understand their impact and to develop appropriate regulatory measures. Analysts have developed a wide array of techniques and technologies to address the ongoing trend of ACH use, both in the laboratory and at crime scenes. Current methodologies are being developed with a focus on sensitivity to correlate drug abuse with clinical cases and practicality, with special attention to sensor development for onsite use. Blood and urine are the most common matrices used to identify ACHs, due to the pharmacokinetics of these drugs, which have a considerable circulation period and are excreted via renal elimination. Alternative matrices are becoming more recurrent as analysis targets, especially hair due to its stability and ease of collection. Oral fluid is less interesting as a matrix for these drugs due to low bioavailability. LLE and SPE remain the most widely used pre-treatment techniques, with the latter still offering the advantage of being a fully automated technique. Nevertheless, miniaturised techniques have gained traction, primarily due to the development of new materials aimed at identifying ACHs or addressing environmental concerns, as most of these techniques align with the principles of green analytical chemistry. In terms of determination methods, chromatography, particularly LC-MS/MS, is the most widely used due to its excellent separation capabilities and high sensitivity. GC-MS is also employed, valued for its cost-effectiveness, although it may require derivatisation for some ACH analogues to improve sensitivity. Immunoassays are beneficial for initial screening due to their ease of use, but they often necessitate more complex techniques for accurate detection and may lack commercial kits for most ACH analogues. Additionally, cross-reactions must be carefully evaluated to avoid false results. Concerning the use of sensors, electrochemical sensors are predominant due to their high sensitivity and capability of integrating new materials as electrodes and sensing layers. However, optical sensors provide better applicability for onsite recognition with post-discrimination using more specific equipment.

Overall, continuous advancements in analytical techniques and regulatory measures are crucial to effectively address the challenges posed by the recreational and medical use of ACHs.

## Abbreviation

2FDCK	2-fluorodeschloroketamine
2FDCNEK	2-fluorodeschloro-n-ethyl-ketamine
2-oxo-PCE	n-ethyl-deschloroketamine
3-MeO-PCE	3-methoxyeticyclidine
3-MeO-PCP	3-methoxyphencyclidine
3-MPA	3-mercaptopropionic
3-OH-PCP	3-hydroxyphencyclidine
4-MeO-PCP	4-methoxyphencyclidine
8BA-2F	((((Hexane-1,6-diylbis(2,7-bis(4-(hydroxymethyl)-phenyl)-9H-fluorine-9,9-diyl))bis(hexane-6,1-diyl))bis-(9H-carbazole-9,3,6-triyl))tetrakis(benzene-4,1-diyl))tetramethanol
ACHs	Arylcyclohexylamines
Cal	Calcein
CD	carbon dots
CEDIA	cloned enzyme donor immunoassay
CE	18-crown-6
CNS	central nervous system
DAD	diode array detector
DBP	dibutyl phthalate
DBS	dried blood spots
DEA	Drug Enforcement Administration
DESI	desorption electrospray ionization
DHNK	Dehydronorketamine
DLLME	dispersive liquid/liquid microextraction
DME	dual mode extraction
DNA	deoxyribonucleic acid
DXE	deschloroketamine
EDC	1-ethyl-3-(3-dimethylaminoprophyl) carbodiimide
EGDMA	ethylene glycol dimethacrylate
EI/CI	ion trap electron and chemical ionization
EIA	enzyme immunoassay
EIS	electrochemical impedance spectroscopy
ELISA	enzyme-linked immunosorbent assays
EMCDDA	European Monitoring Centre for Drugs and Drug Addiction
ESI	electrospray ionization
EU	European Union
FID	flame ionization detector
GC	gas chromatography
HPLC	high pressure liquid chromatography
HRAM	high resolution accurate mass
HRMS	high resolution mass spectrometry
HS	headspace
IMS	ion mobility spectrometry
IT	ion trap
KET	ketamine
KTpClPB	potassium tetrakis(p-chlorophenyl)borate
LC	liquid chromatography
LLE	liquid/liquid extraction
LOD	limit of detection
Lu	luminol
MAA	methacrylic acid
MEPS	microextraction by packed sorbent
MIP	molecularly imprinted polymer
MOFs@G	metal-organic framework/graphene nanocomposite
MS/MS	tandem mass spectrometry
MXE	methoxetamine
MXPr	methoxpropamine
Na-TPB	lipophilic anionic additive
NBS	n-bromosuccinimide
NENK	n-ethyl-norketamine
NHS	n-hydroxysulfsuccinimide
NKET	norketamine
NMDAr	N-methyl-D-aspartate receptor
NMR	nuclear magnetic resonance
NP	nanoparticles
N.S.	non-specified
N.A.	non-applicable
PANI-rGO	polyaniline-reduced graphene oxide
PBI-CB	o-carborane derivative of perylene bisimide
PCE	eticyclidine
PCP	phencyclidine
PCF	photonic crystal fibres
PDMS	polydimethylsiloxane
PGE	pencil graphite electrode
PLE	pressurized liquid extraction
PM-CE	a-keggin polyoxomolybdophosphate:18-crown-6
PM-KT	phosphomolybdic–ketamine
PPT	protein precipitation
PTY	polytyramine
Q8	cucurbit[8]uril
QTOF	quantitative time-of-flight
sd-GC-FTIR	solid deposition gas chromatography Fourier transform infrared spectrometry
SMART	Global Synthetics Monitoring: Analyses, Reporting and Trends
SPE	solid-phase extraction
SPR	surface plasmonic resonance
TA-Ag NCs	thiosalicylic acid-stabilized silver nanoclusters
THz	terahertz
TMSPMA	3-(trimethoxysilyl)propyl methacrylate
TOF	time-of-flight
UHPLC	Ultra-high pressure liquid chromatography
UNODC	United Nations Office on Drugs and Crime
UV	ultra-violet
Zeo–GO	zeolites nanoflakes and graphene-oxide nanocrystal
